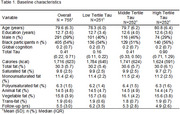# Dietary Fat Intake, Blood Tau Levels, and Cognitive Decline in Older Adults

**DOI:** 10.1002/alz70860_107782

**Published:** 2025-12-23

**Authors:** Xiaoran Liu, Todd Beck, Pankaja Desai, Klodian Dhana, Denis A Evans, Kumar B Rajan

**Affiliations:** ^1^ Rush University Medical Center, Chicago, IL, USA; ^2^ Rush Institute for Healthy Aging, Chicago, IL, USA; ^3^ Rush Alzheimer's Disease Center, Chicago, IL, USA

## Abstract

**Background:**

Previous evidence suggests that different types of dietary fat—including saturated fatty acids (SFA, primarily from animal products) and unsaturated fats (monounsaturated [MUFA] and polyunsaturated fatty acids [PUFA])—may have distinct effects on brain health and cognition. However, the underlying mechanisms remain poorly understood. This study examines the association between dietary fat intake, neuronal cytoskeletal biomarkers (total tau [t‐tau]), and cognition in an aging population cohort.

**Methods:**

Data were analyzed from 755 participants in the Chicago Health and Aging Project (CHAP). Global cognition was assessed using a composite score incorporating episodic memory, perceptual speed, and the Mini‐Mental State Examination (MMSE). Dietary fat intake was measured using a 144‐item food frequency questionnaire and expressed as a percentage of total energy intake. Serum t‐tau levels were quantified using a single‐molecule array (Simoa) bead‐based platform at Quanterix Corporation (Billerica, MA). Longitudinal mixed‐effects regression models were used to examine the associations between dietary fat intake, blood t‐tau levels, and cognitive decline, adjusting for age, sex, education, and total calorie intake.

**Results:**

Participants had a mean age of 79 years (SD = 6.3), with 54% Black and 61% female. The mean follow‐up was 5.5 years. Dietary fat intake as a percentage of total energy was 30.3% for total fat, 9.8% for SFA, 11.4% for MUFA, and 6.3% for PUFA. Mean circulating total tau levels were 0.41 pg/mL (SD = 0.22). Total fat, vegetable fat, and MUFA significantly modified the association between total tau levels and global cognitive decline (*p* for interaction = 0.023, <0.001, and 0.01, respectively). A 1% increase in total fat, vegetable fat, and MUFA was associated with a slower rate of cognitive decline (β = 0.0046 ± 0.02, β = 0.0071 ± 0.0026, and β = 0.013 ± 0.005, respectively), translating to a 9% slower decline for total fat, 12.5% for vegetable fat, and 24% for MUFA. No significant effect modification was observed for SFA or PUFA.

**Conclusions:**

Dietary fat intake significantly modified the association between blood neurodegenerative cytokines and cognition, suggesting that diet‐tau interactions may influence cognitive function in older adults.